# Supplementary material for: Highly Proton-Conducting Membranes Based on Poly(arylene ether)s with Densely Sulfonated and Partially Fluorinated Multiphenyl for Fuel Cell Applications
Source: Membranes (Basel). 2021 Aug 15;11(8):626. doi: 10.3390/membranes11080626 (PMC8398039; doi:10.3390/membranes11080626)
Supplement: Supplementary file 1 [file membranes-11-00626-s001.zip › membranes-1338621-supplementary.pdf]

Supporting Materials

Highly Proton-Conducting Membranes Based on Poly(Arylene Ether)s with Densely Sulfonated and Partially Fluorinated Multiphenyl for Fuel Cell Applications

Tzu-Sheng Huang [1], Tung-Li Hsieh[2], Chih-Ching Lai [1], Hsin-Yi Wen[3], Wen-Yao Huang [1,*] and Mei-Ying Chang [1,*]

1 Department of Photonics, National Sun Yat-Sen University, Kaohsiung 804, Taiwan; zxp86133@gmail.com (T.S.H.); jackko61010@gmail.com (C.C.L.)
2 General Education Center, Wenzao Ursuline University of Languages, Kaohsiung 807, Taiwan; tunglihsieh@gmail.com (T.L.H.)
3 Department of Green Energy and Environmental Resources, Chang Jung Christian University, Tainan City 71101, Taiwan.; hywen@mail.cjcu.edu.tw (H.Y.W.)
* Correspondence: wyhuang@mail.nsysu.edu.tw (W.Y.H.); mychang01@mail.nsysu.edu.tw (M.Y.C.)

We also measured the FTIR, 1H NMR and MALDI-TOF of this series compounds, shown in Figure S1 to Figure S16.

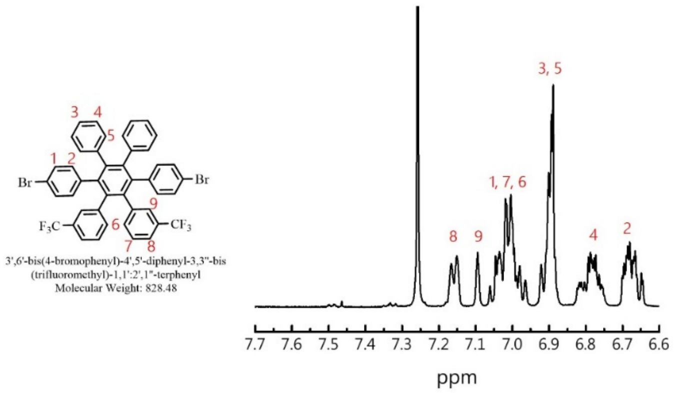

Figure S1. ^{1}H NMR spectrum of 3′,6′-bis(4-bromophenyl)-4′,5′-diphenyl-3,3′′-bis (trifluoromethyl)-1,1′:2′,1′′-terphenyl (1) (6F7B-DB).

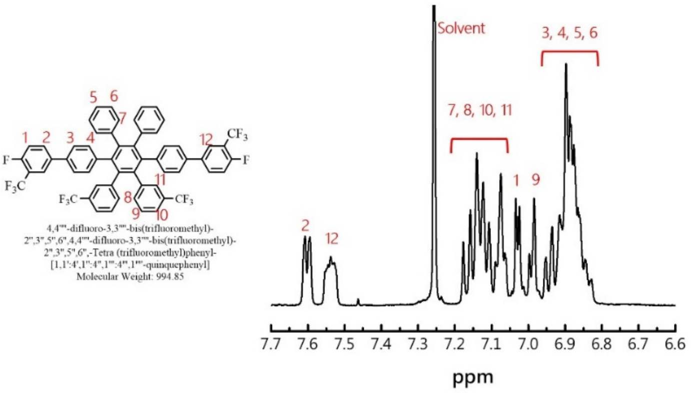

Figure S2. ^{1}H NMR spectrum of 4,4′′′′-difluoro-3,3′′′′-bis(trifluoromethyl)-2′′,3′′,5′′,6′′,4,4′′′′-difluoro-3,3′′′′-bis(trifluoromethyl)-2′′,3′′,5′′,6′′,-Tetra (trifluoromethyl)phenyl-[1,1′:4′,1′′:4′′,1′′′:4′′′,1′′′′-quinquephenyl] (2) (12F9B-DF) .

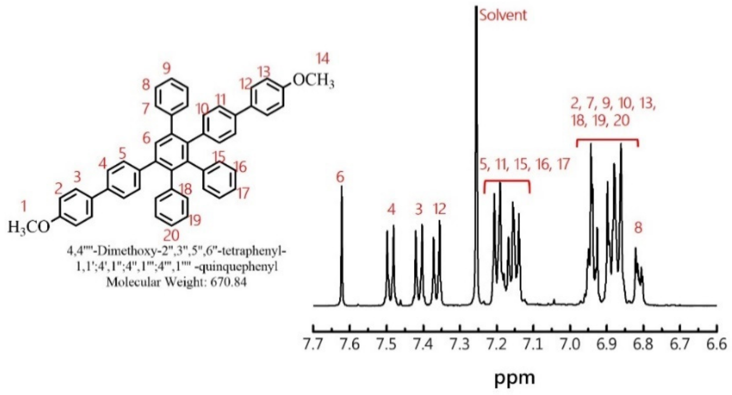

Figure S3. ^{1}H NMR spectrum of 4,4′′′′-Dimethoxy-2′′,3′′,5′′-triphenyl-1,1′:4′,1′′:4′′,1′′′:4′′′,1′′′′-quinquephenyl (3) (8B-DMO).

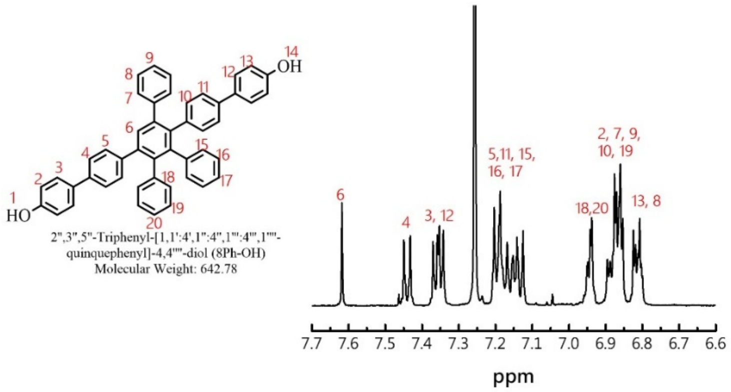

Figure S4. ^{1}H NMR spectrum of 2′′,3′′,5′′-Triphenyl-[1,1′:4′,1′′:4′′,1′′′:4′′′,1′′′′-quinquephenyl]-4,4′′′′-diol (4) (8B-DO).

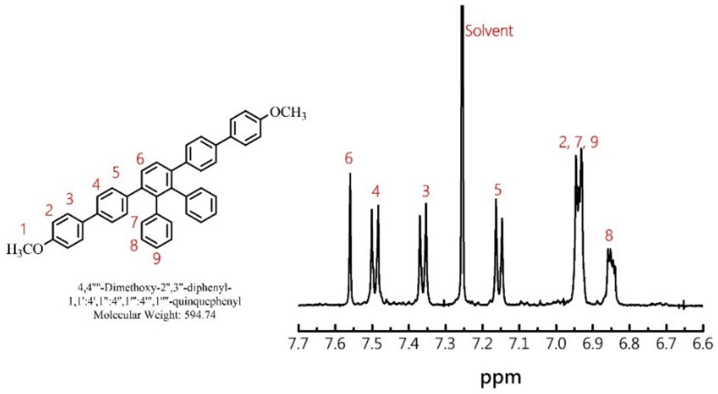

Figure S5. ^{1}H NMR spectrum of 4,4′′′′-Dimethoxy-2′′,3′′-diphenyl-1,1′:4′,1′′:4′′,1′′′:4′′′,1′′′′-quinquephenyl (5)(7B-DMO).

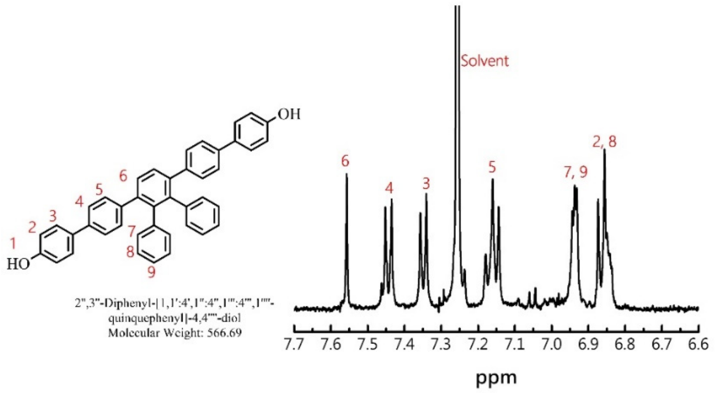

Figure S6. ^{1}H NMR spectrum of 2′′,3′′-Diphenyl-[1,1′:4′,1′′:4′′,1′′′:4′′′,1′′′′-quinquephenyl]-4,4′′′′-diol (6) (7B-DO).

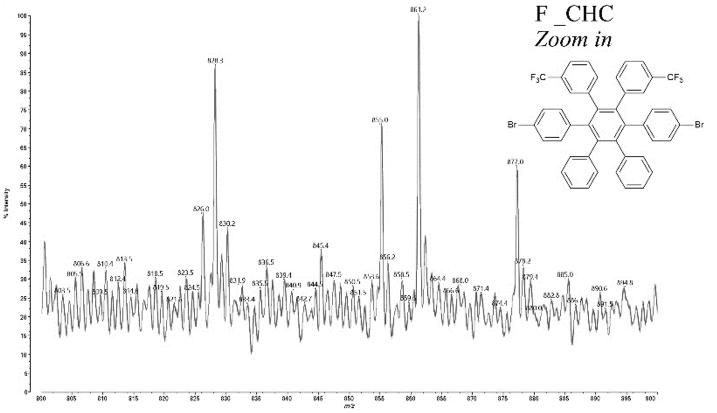

Figure S7. MALDI-TOF spectrum of 6F7B-DB.

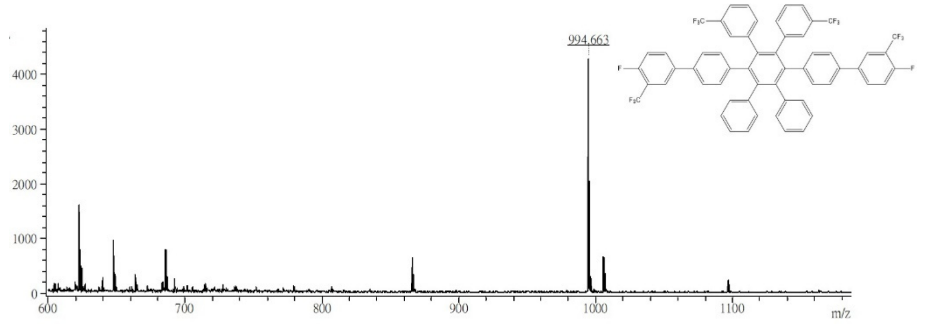

Figure S8. MALDI-TOF spectrum of 12F9B-DF.

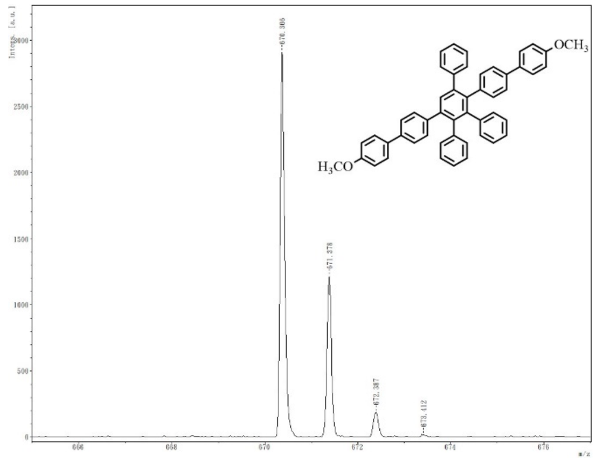

Figure S9. MALDI-TOF spectrum of 8B-DMO.

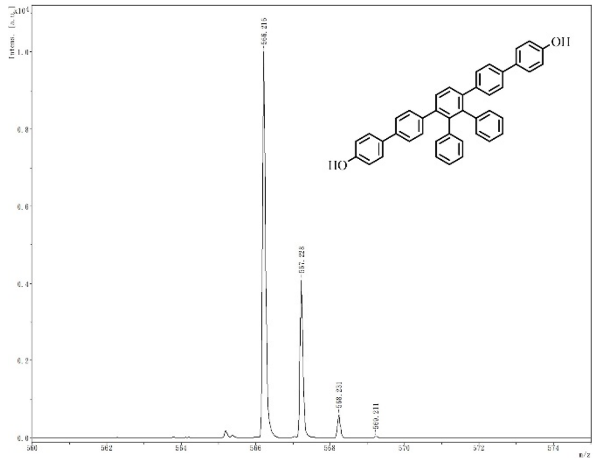

Figure S10. MALDI-TOF spectrum of 8B-DO.

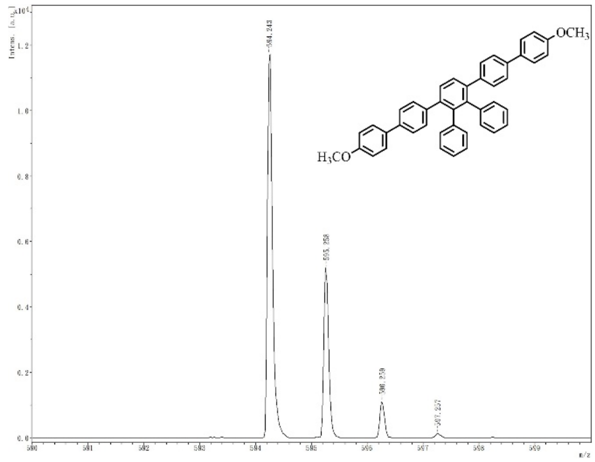

Figure S11. MALDI-TOF spectrum of 7B-DMO.

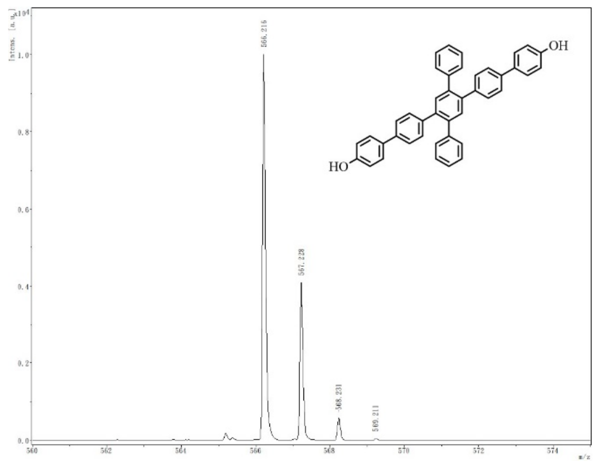

Figure S12. MALDI-TOF spectrum of 7B-DO.

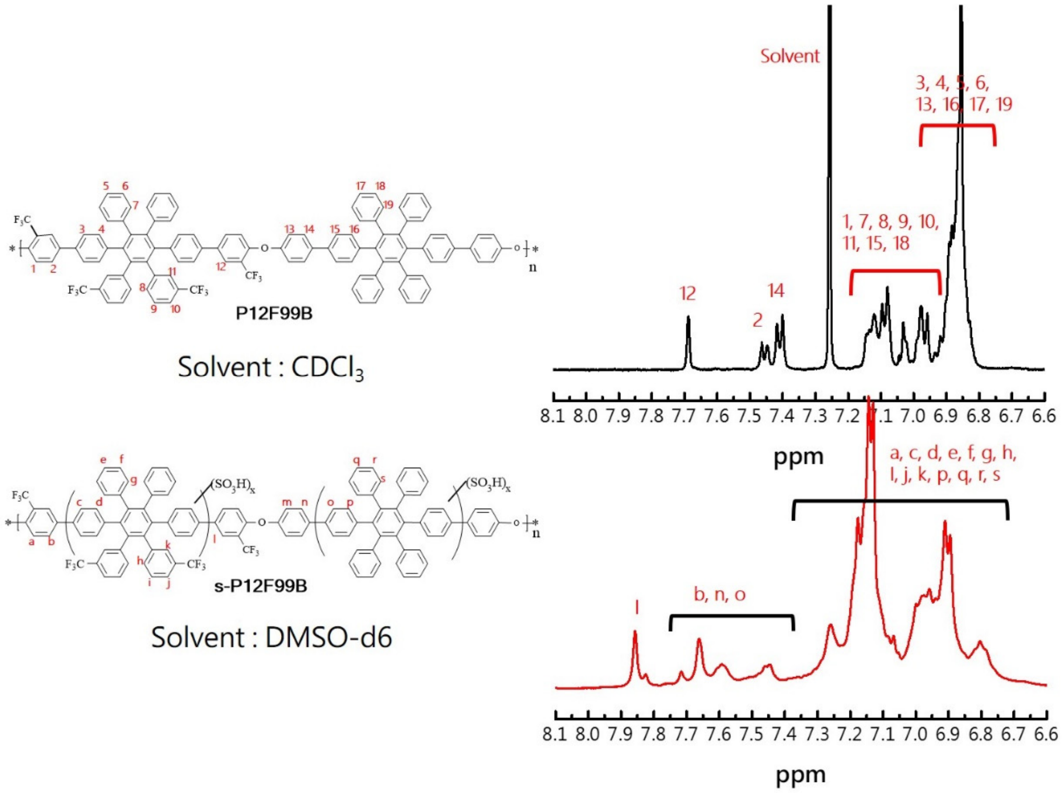

Figure S13. ^{1}H NMR spectrum of P12F99B (top) and s-P12F99B (bottom).

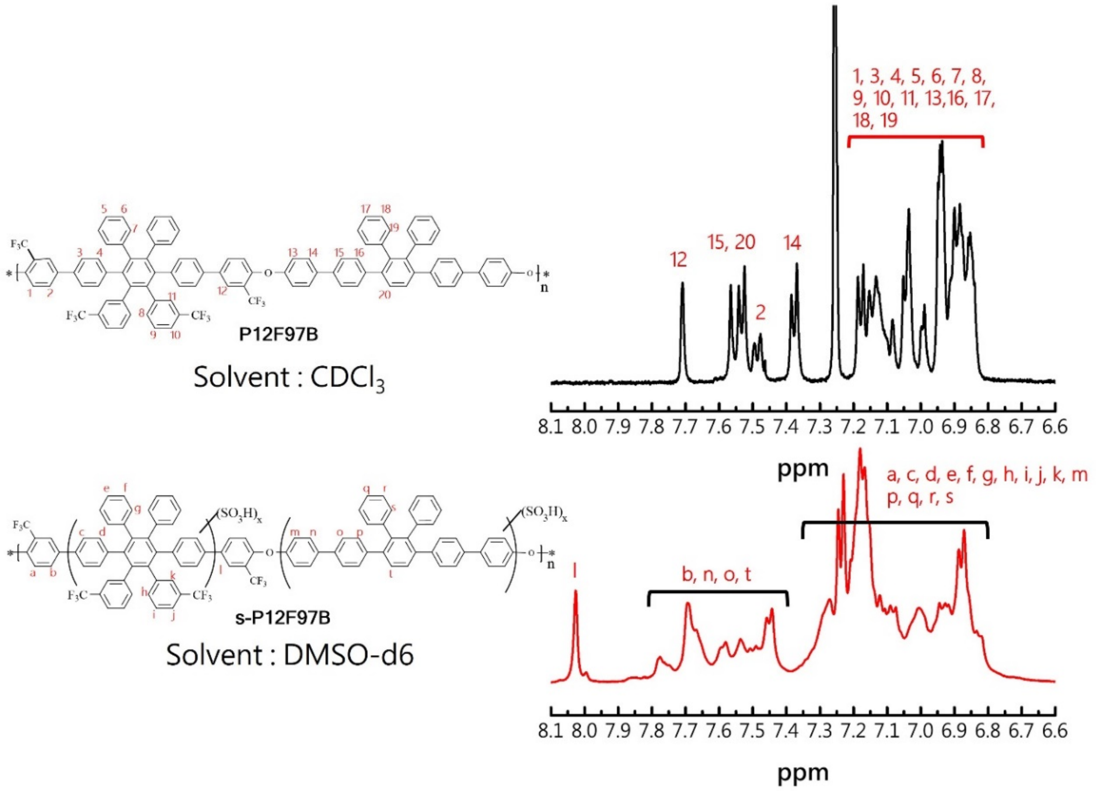

Figure S14. ^{1}H NMR spectrum of P12F97B (top) and s-P12F97B (bottom).

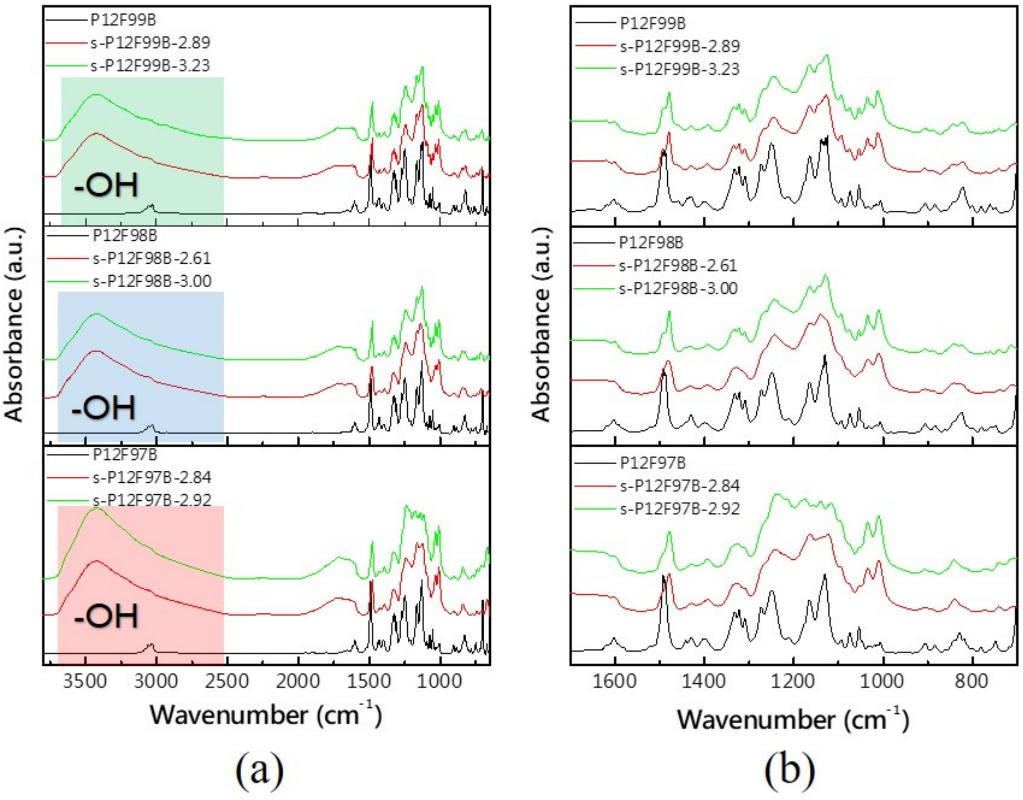

Figure S15. FTIR spectra of the P12FmB polymers and after sulfonation from (a) 650 to 3800 cm^{-1} and (b) 700 to 1700 cm^{-1}.
